# COVID-19 Molecular Pathophysiology: Acetylation of Repurposing Drugs

**DOI:** 10.3390/ijms232113260

**Published:** 2022-10-31

**Authors:** Jong Hoon Lee, Badar Kanwar, Asif Khattak, Jenny Balentine, Ngoc Huy Nguyen, Richard E. Kast, Chul Joong Lee, Jean Bourbeau, Eric L. Altschuler, Consolato M. Sergi, Tuan Ngoc Minh Nguyen, Sangsuk Oh, Mun-Gi Sohn, Michael Coleman

**Affiliations:** 1 Science and Research Center, Seoul National University College of Medicine, 103 Daehak-ro, Jongno-gu, Seoul 03080, Korea; 2 Department of Intensive Care Unit and Neonatal Intensive Care, Hunt Regional Hospital, Greenville, 75401 TX, USA; 3 Department of Health, Phutho Province, Tran Phu Str., Viet Tri City 227, Vietnam; 4 IIAIGC Study Center, Burlington, VT 05408, USA; 5 Department of Anesthesiology, Seoul National University Hospital, Seoul National University College of Medicine, Seoul 03080, Korea; 6 Respiratory Epidemiology and Clinical Research Unit, McGill University Health Centre, Montréal, QC H4A 3S5, Canada; 7 Department of Physical Medicine and Rehabilitation, Metropolitan Hospital, New York, NY 10029, USA; 8 Division of Anatomical Pathology, Children’s Hospital of Eastern Ontario (CHEO), University of Ottawa, 401 Smyth Road, Ottawa, ON K1H 8L1, Canada; 9 General Hospital of Phutho, Nguyen Tat Thanh, Str., Viet Tri City 227, Vietnam; 10 Department of Food Engineering, Food Safety Laboratory, Memory Unit, Ewha Womans University, Seoul 03600, Korea; 11 Department of Food Science, KyungHee University College of Life Science, Seoul 17104, Korea; 12 College of Health and Life Sciences, Aston University, Birmingham B4 7ET, UK

**Keywords:** ACE2 (angiotensin-converting enzyme 2), aspirin, cGAS–STING (cyclic guanosine monophosphate (GMP)-adenosine monophosphate (AMP) synthase (cGAS)–stimulator of interferon genes (STING)), dapsone, dexamethasone, immunologic engram, inflammasome, SAMHD1 (sterile alpha motif and histidine-aspartate domain-containing protein 1), SARS-CoV-2 (severe acute respiratory syndrome coronavirus 2), spike protein, TLR4 (Toll-like receptor 4)

## Abstract

Severe acute respiratory syndrome coronavirus 2 (SARS-CoV-2) induces immune-mediated type 1 interferon (IFN-1) production, the pathophysiology of which involves sterile alpha motif and histidine-aspartate domain-containing protein 1 (SAMHD1) tetramerization and the cytosolic DNA sensor cyclic-GMP-AMP synthase (cGAS)–stimulator of interferon genes (STING) signaling pathway. As a result, type I interferonopathies are exacerbated. Aspirin inhibits cGAS-mediated signaling through cGAS acetylation. Acetylation contributes to cGAS activity control and activates IFN-1 production and nuclear factor-κB (NF-κB) signaling via STING. Aspirin and dapsone inhibit the activation of both IFN-1 and NF-κB by targeting cGAS. We define these as anticatalytic mechanisms. It is necessary to alleviate the pathologic course and take the lag time of the odds of achieving viral clearance by day 7 to coordinate innate or adaptive immune cell reactions.

## 1. Introduction

Severe acute respiratory syndrome coronavirus 2 (SARS-CoV-2) induces immune-mediated inflammasome diseases [1]. The innate immune system is an evolutionarily ancient component of the immune system that is responsible for the rapid detection of potential dangers to the host. It is composed of myeloid effector cells that recognize these dangers through a limited repertoire of germline-encoded receptors that are invariant and recognize conserved microbial patterns and the components associated with cellular damage. It plays an essential role in direct antimicrobial responses and in instructing the development of an adaptive immune response. Several inflammasome activators have been identified comprising: viruses (including influenza virus and adenovirus), bacteria, fungi, bacterial pore-forming toxins, ATP, crystalline particulates, chemical irritants, and ultraviolet B. These induce familial cold autoinflammatory syndrome (FCAS), Muckle–Wells syndrome, neonatal-onset multisystem inflammatory disease (NOMID), gout, pseudogout, silicosis, asbestosis, type II diabetes mellitus, familial Mediterranean fever (FMF), vitiligo-associated multiple autoimmune diseases, Crohn’s disease, and Blau syndrome [2]. Coronaviruses are divided mainly into four genera, α, β, γ, and δ, based on their genomic structure. α and β coronaviruses infect only mammals. Severe acute respiratory syndrome coronavirus (SARS-CoV), Middle East respiratory syndrome coronavirus (MERS-CoV), and SARS-CoV-2 are classified as β coronaviruses [3].

Our current perception of coronavirus disease-2019 (COVID-19) immunopathology does not fully elucidate how the innate and adaptive host immune systems appear to miscommunicate to worsen the viral impact and patient prognosis. The bronchoalveolar immune response has a unique local profile in COVID-19 that strongly differs from the immune profile in peripheral blood: T cell activation in blood, but not in bronchoalveolar lavage fluid (BALF), was found to be higher in fatal COVID-19 cases, and increased levels of inflammatory mediators were more pronounced in BALF than in plasma [4]. Chronic hyperinflammatory monocytes are enriched in critical COVID-19 and contribute to an ATP–purinergic signaling–inflammasome footprint that could enable COVID-19–associated fibrosis and worsen disease severity. Alveolar macrophages are also depleted in critical COVID-19, characterized by anti-inflammatory and antigen-presenting characteristics [5].

The pathophysiologic mechanisms driving multiorgan damage include direct viral infection, maladaptive functions of the renin–angiotensin–aldosterone system (RAAS), impacts on endothelial cell damage and the generation of a thrombotic milieu, dysregulation of the immune response, and cytokine release syndrome. Nonetheless, many of the morphological data collected are nonspecific, variable, and possibly associated with other coexisting factors, such as the mechanisms contributing to endothelial–epithelial barrier breakdown during the monocyte and neutrophil invasion process [6]. The extravasation of a protein-rich exudate into the alveolar space is consistent with other forms of acute respiratory distress syndrome (ARDS) [7]. The pathology of ARDS thrombosis is unclear compared with platelets for acute lung injury caused by other respiratory pathogens (bacteria, viruses, plasmodia) [8]. Angiotensin-converting enzyme 2 (ACE2) is upregulated in the lung tissue and serum of COVID-19 ARDS and unrelated ARDS, whereas a loss of type 2 alveolar epithelial (AT2) cells is selectively observed in COVID-19 ARDS. A decrease in AT2 cells may distinguish COVID-19 from unrelated ARDS, whose underlying mechanism may play critical roles in post-injury repair and possibly involves an increased risk of pulmonary fibrosis [9]. Regardless of the variation in the anti-inflammatory response, pulmonary infection and injury are also associated with systemic inflammation and sepsis [10].

Innate immune cells, including macrophages, monocytes, dendritic cells, neutrophils, and innate lymphoid cells (ILCs) such as natural killer (NK) cells, recognize pathogen-associated molecular patterns (PAMPs) or damage-associated molecular patterns (DAMPs) to induce inflammatory signaling pathways and immune responses. They are armed with an arsenal of five pattern recognition receptors (PRRs) that include Toll-like receptors (TLRs), retinoic-acid-inducible gene I (RIG-I)-like receptors (RLRs), nucleotide-binding oligomerization domain (NOD)-like receptors (NLRs), C-type lectin receptors, and absent in melanoma 2 (AIM2)-like receptors [11]. Several PRRs of TLRs, RLRs, NLRs, and inflammasomes have activated their signaling pathways in response to SARS-CoV-2 [12]. Therefore, we need medicine to prevent these signaling pathways.

## 2. Molecular Pathophysiology

### 2.1. SAMHD1 Tetramerization Yields the Catalytically Active Tetramer: SARS-CoV-2 Might Use CDK2 to Phosphorylate SAMHD1

The sterile alpha motif (SAM) is a protein interaction domain involved in developmental regulation. SAM is an evolutionarily conserved protein-binding domain that regulates numerous developmental processes among diverse eukaryotes [13]. SAM and histidine-aspartate domain (HD)-containing protein 1 (SAMHD1) deplete the pool of deoxynucleoside triphosphates (dNTPs) available to a reverse transcriptase for viral cDNA synthesis and thus prevent viral replication [14]. SAMHD1 protects cells from viral infections and operates at stalled replication forks to prevent IFN induction, a significant regulator of dNTP concentrations in human cells [15]. SAMHD1 is a cellular enzyme that drains intracellular dNTPs.

SAMHD1 can restrict retroviruses (human immunodeficiency virus-1, HIV-1). Mutations in SAMHD1 are linked to the pathogenesis of chronic lymphocytic leukemia and Aicardi–Goutières syndrome (AGS). SAMHD1 holds a target motif for cyclin-dependent kinase 1 (CDK1), whose activity is needed for SAMHD1 phosphorylation. SAMHD1 is phosphorylated at residue threonine 592 (T592) in cycling cells, and phosphorylated SAMHD1 on T592 cannot block retroviral infection. Phosphorylation modulates SAMHD1 to block retroviral infection without affecting its ability to decrease cellular dNTP levels [16]. A phosphomimetic mutation surrounding T592 triggered electrostatic repulsion from a distinct negatively charged environment. This resulted in a significant decrease in active SAMHD1 tetramers; hence, the dNTPase activity was substantially decreased. SAMHD1 phosphorylation at residue T592 may modulate its cellular and antiviral functions [17].

Apoenzymes are catalytically inactive, whereas holoenzymes are catalytically active. Inactive apo-SAMHD1 interconverts between monomers and dimers. The binding of deoxyguanosine triphosphates (dGTP) to four allosteric sites promotes tetramerization. It induces a conformational change in the substrate-binding pocket to yield the catalytically active tetramer [18]. The binding sites are plastic, and the allosteric binding sites can adjust oligonucleotides in place of the allosteric activators GTP and dNTP. The binding of G-nucleotide-containing oligonucleotides in the presence of GTP and dNTPs promotes the formation of a specific tetramer with mixed occupancy of the allosteric sites responsible for the antiretroviral activity of SAMHD1 [19].

### 2.2. cGAS–STING Signaling

The SAMHD1 tetramer structure could provide a mechanistic understanding of its rapid function in SARS-CoV-2. SAMHD1 negatively regulates the interferon (IFN)-1 signaling pathway: the elevated innate immune response and IFN activation upon genetic loss of SAMHD1 effectively suppress SARS-CoV-2 replication [20]. The spontaneous IFN response requires the cyclic guanosine monophosphate (GMP)-adenosine monophosphate (AMP) synthase (cGAS)–stimulator of interferon genes (STING) cytosolic DNA-sensing pathway in SAMHD1-deficient cells in mice. SAMHD1 mutations cause autoinflammatory AGS, characterized by chronic type I IFN secretion in the absence of infection with exogenous viruses [21] and typified by early onset brain disease [22]. IFN gamma (γ) is a dimerized soluble cytokine, and its signaling is upregulated in the COVID-19 human neurovascular unit [23]. SARS-CoV-2 evokes a response that requires the strong induction of a subclass of cytokines, including type I and, obviously, type III IFN and a few chemokines, such as those produced in response to influenza A virus and, in particular, respiratory syncytial virus [15,24]. SAMHD1 limits the virus-induced production of IFNs and the induction of costimulatory markers during lentivirus infection in myeloid cells. This programmed myeloid cell activation requires reverse transcription, cGAS–STING, and signaling via the interferon receptor. SAMHD1 reduced the stimulation of virus-specific cytotoxic T cells in vivo and limited the induction of antigen-specific T cell responses in vivo. SAMHD1 controls viral infection through innate and adaptive immunity at the level of the infected cell [25].

**Figure 1:**
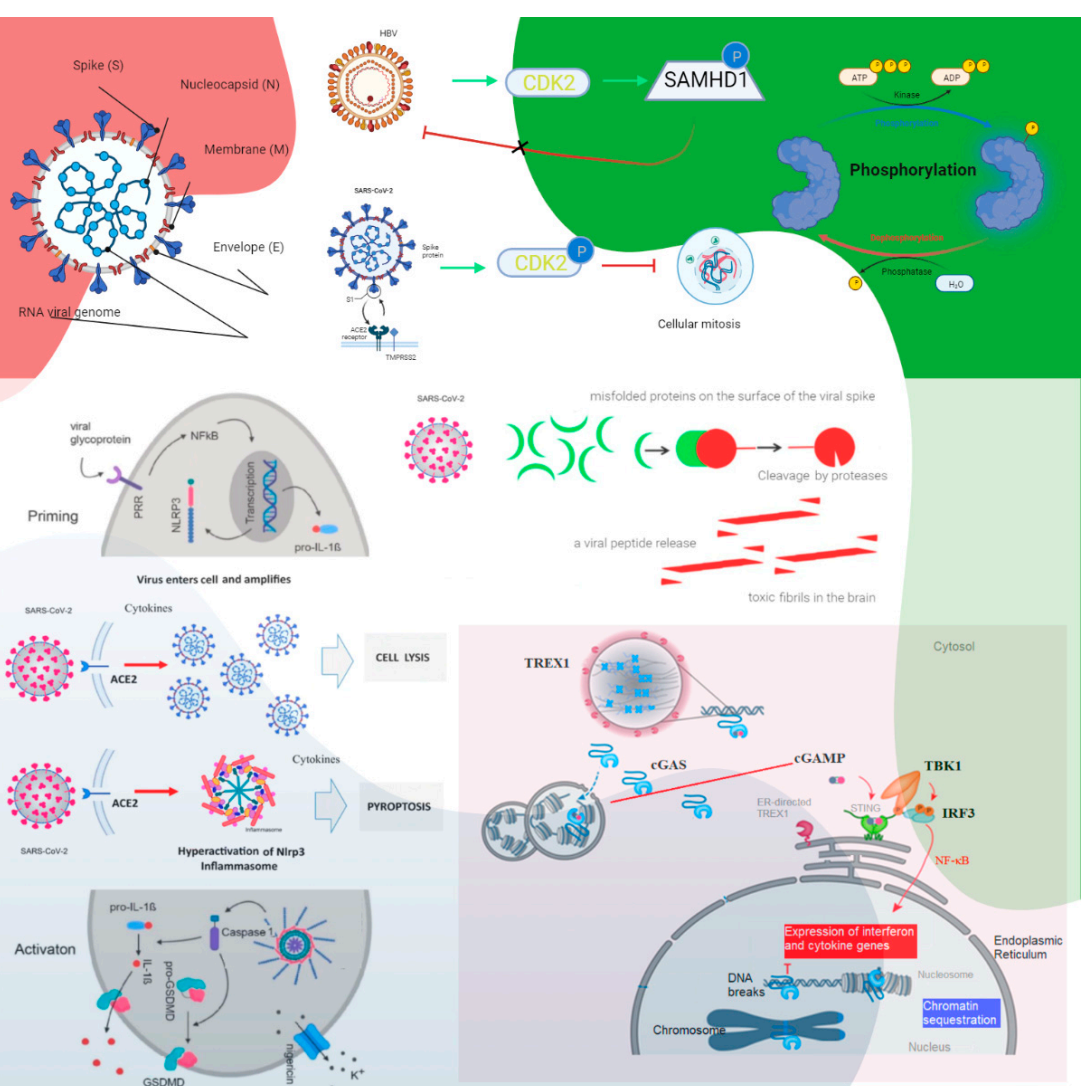
The roles of cyclin-dependent kinase and cGAS–STING signaling. Cyclin-dependent kinase 1 (CDK1), CDK2, and CDK6 inhibit SAMHD1 antiviral function via phosphorylation and inactivation. HBV recruits cyclin E2 to bind CDK2 and further phosphorylates SAMHD1 to abrogate its restriction of HBV replication. SARS-CoV-1 suppresses the activity of cyclin d-CDK4 and cyclin A/E–CDK2 complexes, and SARS-CoV-2 enhances the phosphorylation of CDK2 to inhibit cellular mitosis. Pattern recognition receptors are essential in sensing pathogen-associated molecular patterns (PAMPs) and DAMPs. For example, Toll-like receptors (TLRs) recognize a variety of PAMPs and DAMPs that initiate the inflammatory process via NF-κB (nuclear factor-kappa B cells) and the synthesis and release of cytokines and IFNs. Inflammasomes are a separate class of intracellularly expressed pattern recognition receptors (PRRs) that recognize nucleic acids and mediate proinflammatory responses. Cell surface–expressed ACE2 and TLR4 increase the NLRP3 inflammasome downstream mediator caspase-1, and exposure to spike proteins upregulates the protein expression involved in the positive stimulation of TLR4 signaling and the inflammasome pathway. The induction of neuroinflammation in microglia is mediated through the activation of NF-κB and p38 MAPK (mitogen-activated protein kinase), possibly due to TLR4 activation. TLR4 has been found to play a critical role as a mediator of the neurotoxicity induced by α-synuclein oligomers. Misfolded α-synuclein (α-Syn) induces inflammatory responses, and extracellular α-Syn can activate proinflammatory TLR4 pathways in astrocytes, but α-Syn uptake is independent of TLR4. The interaction between TLR4 and the SARS-CoV-2 spike protein can trigger an intracellular TLR4 signaling cascade. NF-kB’s transcriptional activation of specific genes induces the release of proinflammatory cytokines, which can cause neuronal damage and the pathological modification of α-Syn. Serum neurofilament light chain (NFL) is a biomarker of neuronal injury. NFL was higher in COVID-19 patients than in the control groups. Higher NFL levels were associated with neuronal injury. This is common in critically ill patients. The hyperinflammatory state of COVID-19 has high levels of proinflammatory cytokines, and this might have triggered central nervous system neuroinflammation through the activation of astrocytes and microglia, which could have facilitated prion-like pathology. In addition, similar to other prion proteins, the spike protein also contains several prionogenic domains. Thus, direct toxic action of the spike protein, triggering a neurodegenerative condition mimicking a prion disease-like pathology, is also possible. The biological implications of nuclear cGAS and its interaction with chromatin, including various mechanisms for nuclear cGAS inhibition, the release of chromatin-bound cGAS, the regulation of different cGAS pools in the cell, and chromatin structure/chromatin protein effects on cGAS activation, could lead to cGAS-induced autoimmunity. Cytosolic DNA recognition leads to active cGAS by clustering and forming large liquid—liquid phase-separated cGAS–DNA condensates, excluding the ER-directed exonuclease three-prime repair exonuclease 1 (TREX1). Nuclear cGAS is sequestered at chromatin in an inactive state. Active cGAS produces cyclic GMP-AMP (cGAMP), which binds to STING. STING relocalizes to the perinuclear Golgi and forms a clustered platform where the tank-binding kinase 1 (TBK1) kinase phosphorylates the transcription factor IRF3 (interferon regulatory factor 3). Phosphorylated IRF3 enters the nucleus and, along with NF-κB, triggers the expression of type I interferon and proinflammatory cytokine genes (reproduced from [27]) (HBV, hepatitis B virus; P, phosphate; the green arrows indicate enhancement and the dashed arrow indicates the effect is uncertain; CDK, cyclin-dependent kinase; SAMHD1, sterile alpha motif histidine-aspartic acid domain-containing protein 1).

cGAS catalyzes the production of cyclic guanosine monophosphate-adenosine monophosphate (cGAMP) upon sensing cytosolic DNA, which activates STING–tank-binding kinase 1 (TBK1)–interferon regulatory factor 3 (IRF3) signaling. cGAS is present in the nucleus, and nuclear cGAS is sequestered at chromatin in an inactive state. It recruits protein arginine methyltransferase 5 (Prmt5), facilitating IRF3 access upon viral infection. Nuclear-localized cGAS, in innate immunity, interacts with Prmt5 to catalyze the symmetric dimethylation of histone H3 arginine 2 at IRF3-responsive genes (*Ifnb* and *Ifna4)* [26]. Active cGAS produces cyclic GMP-AMP (cGAMP), which binds to STING. STING relocalizes to the perinuclear Golgi and forms a clustered platform where the TBK1 kinase phosphorylates the transcription factor IRF3. Phosphorylated IRF3 enters the nucleus and, along with the nuclear factor kappa-light-chain-enhancer of activated B cells, triggers the expression of type I IFN and proinflammatory cytokine genes [27]. cGAS–STING has been identified as a significant nucleic acid recognition gene. cGAS typically resides as an inactive protein in the cell and is activated upon binding to aberrant DNA. Activated cGAS then synthesizes 2′,3′-cGAMP, which acts as a secondary messenger activating STING. cGAS–STING responds to foreign DNA from viruses and bacteria and to mitochondrial and genomic self-DNA, which enters the cytosol from senescent or dying cells [28]. A SARS-CoV-2 infection could induce syncytia formation within cells expressing ACE2 and the SARS-CoV-2 spike protein, producing micronuclei at an average rate of approximately four per syncytium (>93%). These micronuclei are expressed with a high activation level for the DNA damage response and cGAS–STING signaling. These signaling pathways are associated with cellular catastrophe and aberrant immune activation at the cellular and molecular levels. Activated STING triggers membrane permeabilization and thus lysosomal cell death [29] (Figure 1).

The activation of cGAS–STING can trigger IRF3-type I IFN and autophagy-mediated antiviral activity. SARS-CoV-2 ORF3a can block viral evasion of the STING-triggered autophagy-mediated antiviral function. It can interact with STING and disrupt the STING-light chain 3 (LC3) interaction but not IRF3-type I IFN induction [30]. SARS-CoV-2 infection also activates cGAS–STING signaling by stimulating micronuclei formation during the process of syncytia, and SARS-CoV-2 open reading frame 10 (ORF10) targets STING to antagonize IFN activation. Its overexpression inhibits cGAS–STING-induced IRF3 phosphorylation, translocation, and subsequent IFN induction. In addition, it interacts with STING, attenuates the STING–TBK1 association, and impairs STING oligomerization and aggregation and STING-mediated autophagy. As a result, it prevents the endoplasmic reticulum (ER)-to-Golgi trafficking of STING by anchoring STING in the ER [31].

Profiling skin manifestations, a STING-dependent type I IFN signature is mediated by macrophages adjacent to areas of endothelial cell damage, and cGAS–STING activity was detected in lungs from COVID-19 patients with prominent tissue destruction, which was associated with IFN-1 responses. A lung-on-chip model with SARS-CoV-2 infection activates cGAS–STING signaling in endothelial cells through mitochondrial DNA release. A STING inhibitor shows anti-inflammatory potential by alleviating detrimental immune responses [32]. Among patients with mild disease in a second phase 2 trial between Peginterferon Lambda-1a and placebo groups, it improved symptoms or other clinical metrics of the odds of achieving viral clearance by day 7 [12].

### 2.3. Immunological-Induced Engram Pathway

**Figure 2:**
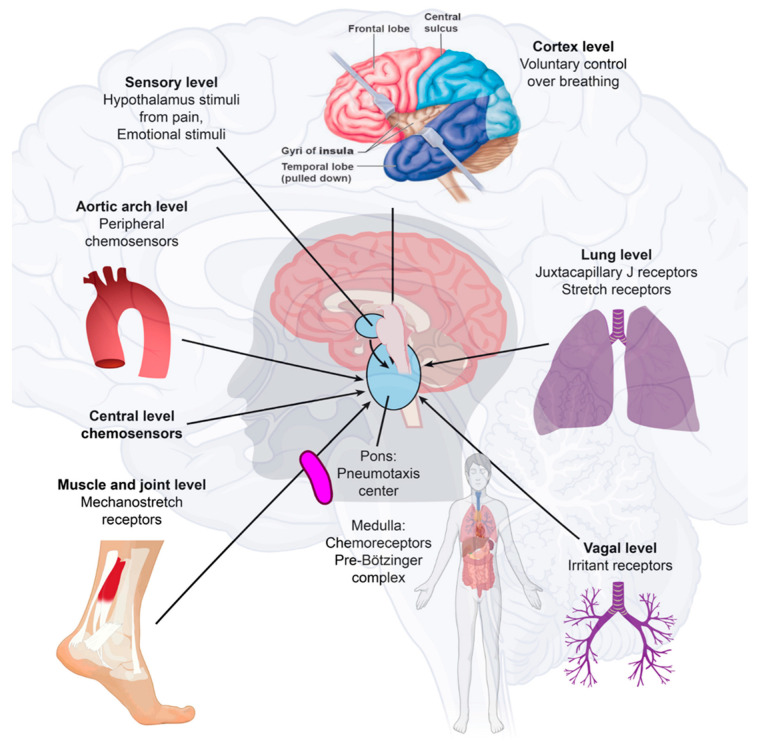
Immune-to-brain communication. The perivascular space contains a single or double layer of invaginated pia in the brain, forming an interstitial fluid-filled space representing an extension of the extracellular fluid space around the intracranial vessels as they move down into the brain parenchyma. Human sensory stimuli affect the breathing sensation passing through the cerebral cortex and hypothalamus. The respiratory muscles are not purposely activated in healthy breathing. Neuropilin-1 (a pleiotropic single-transmembrane coreceptor for class-3 semaphorins and vascular endothelial growth factors) has been confirmed as a coreceptor that facilitates SARS-CoV-2 infection into cells and may be expressed in the brainstem. Activated microglia were observed in the olfactory bulb, midbrain (particularly in the substantia nigra), hindbrain, dorsal motor nucleus of the vagus nerve, and pre-Bötzinger complex in the medulla. Neuroinflammation with microgliosis and T-cell infiltration in COVID-19 brains was significantly greater than that in patients who did not have COVID-19. This might trigger inflammasomes and pyroptosis in the CNS. The pre-Bötzinger complex involvement in the brainstem could account for the respiratory failure and sudden high death rate of COVID-19 ARDS patients. The neurovirulent potential of SARS-CoV-2 is not restricted to patients with mild or severe diseases that can develop neurological complications. Microglia and astrocytes play specific and dynamic roles during immune activation. This immune-to-brain communication occurs when glia, microglia, and astrocytes interpret and propagate inflammatory signals in the brain, and influence physiological and behavioral-change responses. Activating the peripheral immune system with a coordinated brain response elicits and influences physiological and behavioral responses in the immunological memory engram pathway.

SARS-CoV-2 may move from the periphery into the CNS through neurons or the vagus nerve from the lungs or gut [33,34]. In addition, SARS-CoV-2 may disrupt the blood–endothelial barrier by damaging the choroid plexus epithelium due to cytokine storms and systemic inflammation through neuronal cell-surface receptors [23,35] (Figure 2).

Different cell types, including dopaminergic neurons, cortical neurons, brain microvascular endothelial cells, and choroidal epithelial cells, are susceptible to SARS-CoV-2, but there are differences in permissiveness. SARS-CoV-2 is neurovirulent, not restricted to patients with mild or severe diseases, and can cause the development of neurological complications. We found activated microglia in the olfactory bulb, midbrain (specifically, in the substantia nigra), hindbrain, the dorsal motor nucleus of the vagus nerve, and pre-Bötzinger complex on the medulla [36]. Clinically, COVID-19 ARDS can present with relatively preserved aeration on chest computed tomography (CT) imaging despite severe respiratory hypoxemia. However, this early, high-compliance phenotype evolves into a low-compliance phenotype with poor aeration in some patients. We described the first clinical presentation of the L-type as having low elastance (high compliance), low lung weight, low lung recruitment ability, low deranged ventilation–perfusion ratio (VA/Q), and preserved aeration, and the second H-type as a severe ARDS-like picture of hypoxemia, bilateral infiltrates, and increased lung weight. On CT scans, these patients have higher lung weight, high elastance (low compliance), and a high response to PEEP, meaning that the amount of nonaerated lung tissue is very high [37]. COVID-19 patients with L-type respiratory failure exhibit poor oxygenation but have high minute ventilation. Infiltrates on CT scanning have a ground glass appearance that implies interstitial edema rather than alveolar edema [38]. These patients are not overtly dyspneic despite an oxygen saturation of below 90%. COVID-19 causes unique lung injury of the L-type or H-type and different ventilatory approaches are required to treat the underlying physiology [39]. Neuroinflammation with microgliosis and T-cell infiltration in COVID-19 brains was significantly greater than in patients who did not have COVID-19 [39]. We could explain respiratory failure by brainstem involvement with pre-Bötzinger complex involvement and the sudden death of COVID-19 ARDS patients [40,41]. This could explain the L-type rapid respiratory distress and sudden death. COVID-19 may stabilize with the engram pathway at this point without further deterioration.

In many cases, there is no direct virus attack on vulnerable structures. Instead, the neurological disease manifestations may be due to an immune reaction against the virus, and pre-existing neurological disease may become clinically evident or worsen with COVID-19, which explains why various nervous system manifestations react favorably to immune suppression or immune modulation [38,42]. The insular cortex stores immune-related pieces of information. The chemogenetic reactivation of these neuronal bands was sufficient to broadly retrieve the inflammatory state of these neurons depicted in the insular cortex [43]. These immunological memory engrams, as memory traces, can restore the initial disease state [44]. The brain remembers immune challenges, regulates peripheral immunity, and can exacerbate the process of immune reactions [45]. Microglia and astrocytes play specific and dynamic roles during immune activation. This immune-to-brain communication occurs when glia, microglia, and astrocytes interpret and propagate inflammatory signals in the brain and influence physiological and behavioral-change responses. Astrocytes can develop an immunosenescent profile with age. Astrocyte immunosenescence and deficits in IL-10 signaling in the aged brain disrupt the regulation of microglia following innate immune activation [46,47].

## 3. Acetylation and Molecular Treatment Pathway

**Figure 3:**
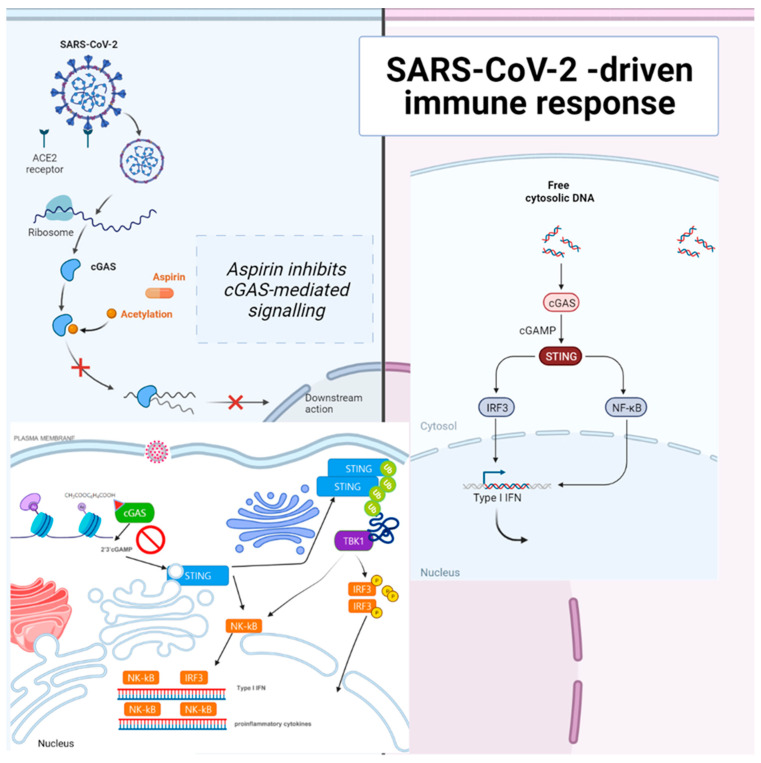
Aspirin strongly inhibited cGAS-mediated TBK1–IRF3 signaling. The sensing of cytosolic DNA by cGAS is central to the pathogenesis of several autoinflammatory syndromes and some autoimmune diseases, such as systemic lupus erythematosus (SLE), because the presence of DNA in the cytoplasm is usually a sign of microbial infections and is quickly detected by cGAS, eliciting anti-infection immune responses. However, the chronic activation of cGAS by self-DNA leads to severe autoimmune diseases for which no effective treatment is yet available. The activation of cGAS signaling requires its deacetylation, while acetylation inhibits cGAS activation, and the compulsory acetylation of cGAS by aspirin forcefully suppresses self-DNA–induced autoimmunity, giving aspirin therapeutic potential in this context. Recently, the unique role of the cGAS–stimulator of the STING pathway in orchestrating innate and adaptive immunity has led to vast interest in employing antagonists of the cGAS–STING pathway as SARS-CoV-2 therapeutic adjuvants. The DNA sensor cGAS detects pathogenic or cytosolic nucleic acids and produces the second messenger, 2′3′-cGAMP, which binds to STING. Activated STING dimerizes and translocates from the endoplasmic reticulum (ER) to the Golgi apparatus. STING is ubiquitinated at the Golgi apparatus, an anchor for TBK1. Stimulation of the cGAS–STING pathway by cytosolic nucleic acids can activate interferon regulatory factor 3 (IRF3) and nuclear factor-κB (NF-κB), and promote the transcription of type I interferons (IFNs) and other proinflammatory cytokines, modulating antigen presentation and immune responses. Therefore, cGAS–STING antagonists are promising adjuvants for constructing effective SARS-CoV-2 therapeutics. Acetylation inhibits cGAS activation, and enforced acetylation of cGAS by aspirin robustly suppresses self-DNA–induced autoimmunity. Aspirin inhibited cGAS-mediated IFN production [39]. The acute or chronic use of ibuprofen and other NSAIDs was not associated with worse COVID-19 disease outcomes [50]. NSAID use might confer a modest benefit concerning survival [51]. Early aspirin prescription may be associated with lower odds of in-hospital mortality among hospitalized patients with moderate COVID-19 in 2,446,650 COVID-19–positive patients [52]. Aspirin is associated with decreased mechanical ventilation, ICU admission, and in-hospital mortality in hospitalized COVID-19 patients. NSAID use might confer a modest benefit concerning survival [51].

Early in the COVID-19 pandemic, there was concern that non-steroidal anti-inflammatory drugs (NSAIDs) could be related to adverse cardiovascular and pulmonary outcomes. Some suggested potential harm with NSAID prescription for COVID-19 patients [48]. However, aspirin inhibited cGAS-mediated IFN production and alleviated DNA-induced autoimmunity in AGS mouse models and patient cells [49]. Aspirin strongly inhibited cGAS-mediated TBK1–IRF3 signaling and did not affect STING activator cGAMP- or cyclic di-GMP (c-di-GMP)-induced downstream TBK1–IRF3 signaling. Aspirin does not inhibit STING-associated vasculopathy with onset in infancy (SAVI)-associated STING mutant-mediated IRF3 activation. Aspirin inhibits the DNA–cGAS–STING–TBK1–IRF3 axis through the inhibition of cGAS [49]. The COVID-19 treatment mechanism of aspirin and dapsone is through SAMHD1 tetramerization and the cGAS–STING signaling pathway (Figure 3).

### 3.1. Aspirin Inhibited cGAS and Did Not Affect STING Directly

Aspirin inhibited cGAS-mediated interferon production and alleviated DNA-induced autoimmunity in AGS mouse models and patient cells. However, another specific COX inhibitor, diclofenac, did not affect cGAS activation [49]. cGAS acetylation on Lys384, Lys394, or Lys414 contributes to keeping cGAS inactive. Aspirin can quickly acetylate cGAS and powerfully inhibit cGAS-mediated immune responses [49,53]. Aspirin can effectively suppress self-DNA–induced autoimmunity in AGS patient cells and an AGS mouse model. Acetylation contributes to cGAS activity control and provides a potential therapy for treating DNA-mediated autoimmune diseases [49]. cGAS activates downstream IRF3 and nuclear factor-κB (NF-κB) signaling via STING [54], and both are involved in IFN production [55]. NF-κB can adjust the expression of some interferon-stimulated genes (ISGs) [56]. Aspirin may deter the activation of both IRF3 and NF-κB by targeting cGAS as a definite cGAS inhibitor.

Aspirin strongly inhibited cGAS-mediated TBK1–IRF3 signaling. However, aspirin does not affect STING activator cGAMP- or cyclic di-guanosine monophosphate (c-di-GMP)-induced downstream TBK1–IRF3 signaling and does not inhibit STING-associated vasculopathy with onset in infancy (SAVI)-associated STING mutant-mediated IRF3 activation. Aspirin inhibits the DNA–cGAS–STING–TBK1–IRF3 axis by inhibiting cGAS [49]. With an acetyl-lysine-incorporating protein synthesis system, Chen et al. created recombinant acetylated cGAS proteins. They showed that the acetylation of these residues strongly inhibits the activity of cGAS and that these acetylation sites participate in the DNA binding of cGAS. The DNA-binding activity of cGAS is slightly reduced by acetylation on each of the individual sites for recombinant acetylated cGAS proteins, consistent with a previous study showing that mutations on some of these residues have only a limited influence on the DNA binding of cGAS. There are six lysine (K) acetylation sites for cGAS: K7, K50, K384, K392, K394, and K414. When K384, K394, or K414 is acetylated, other regions of cGAS, such as the N-terminal region, could still contribute to DNA binding. Several regions of the cGAS protein are essential for cGAS–DNA binding, and the binding of DNA to cGAS leads to the deacetylation of cGAS, promoting cGAS activation. K384 and K414 of cGAS are critical residues for cGAS regulation by multiple post-translational modifications (PTMs). cGAS undergoes K48-linked ubiquitination at K414, leading to the p62-dependent selective autophagic degradation of cGAS in resting cells, and upon DNA-virus infection, this inhibitory ubiquitination is removed, promoting cGAS activation [49,55]. The histone deacetylase (HDAC)-mediated removal of K384 acetylation may allow K27-linked ubiquitination on K384 to further promote cGAS activation [49]. Acetylation on K414 may compete with ubiquitination to maintain an adequate level of cGAS protein in resting cells, and K27-linked ubiquitination on K384 of cGAS has been shown to potentiate cGAS activity in response to DNA-virus infection [57].

### 3.2. Aspirin Treatment Decreased Mortality

Patients progress rapidly to acute respiratory distress syndrome (ARDS), and in the underlying form, septic shock, irreversible metabolic acidosis, blood coagulation dysfunction, and hemostatic and thrombotic anomalies are the leading causes of death due to COVID-19. Platelets play a crucial role in developing severe disease cases. They are the enucleated cells responsible for thrombus formation and the hyper-reactivity induced by proinflammatory microenvironments in the more aggressive course of COVID-19 [58]. Disseminated intravascular coagulation (DIC) may upregulate coagulation pathways by activating procoagulant factors such as tissue factors, leading to arterial and venous thrombotic disease [59]. This high cytokine level is critical in the morbidity and mortality of COVID-19 patients, and cytokine storm syndrome contributes to the upregulation of metabolic coagulation pathways, damaging the endothelium and the cardiovascular system [60]. The antithrombotic properties of aspirin make it a plausible drug for preventing thrombotic disease because aspirin inhibits the platelets and inflammatory cytokines that lead to pathologic platelet aggregation. As a result, aspirin can prevent clot formation and reduce the risk of deep vein thrombosis by 34%, without significantly increasing the risk of bleeding, but not the thrombolysis of an existing clot [61]. Aspirin can modulate multiple pathogenic mechanisms implicated in developing multiple organ dysfunction in sepsis and ARDS [62]. A water-soluble salt of acetylsalicylic acid and two amino acids, glycine and lysine (licensed as aspirin iv.^®^, Tamiflu^®^), was shown to inhibit NF-κB activation and alleviate influenza symptoms in hospitalized patients [63].

There are many aspirin contraindications to consider, such as in DIC and other bleeding disorder settings. Aspirin use can result in uncontrolled hemorrhage and poses a risk of Reye’s syndrome in children. However, we found no evidence of a harmful effect of NSAIDs on COVID-19-related deaths. The risks of COVID-19 do not need to influence decisions about the therapeutic use of NSAIDs [64]. In this cohort of COVID-19 patients, ibuprofen use was not associated with worse clinical outcomes [65]. NSAID and angiotensin-converting enzyme inhibitor (ACE-I)/angiotensin receptor blocker (ARB) use prior to admission is not associated with renal failure or increased mortality [66]. The acute or chronic use of ibuprofen or other NSAIDs was not associated with worse COVID-19 disease outcomes [50]. NSAID use might confer a modest benefit concerning survival [51]. NSAIDs were not associated with 30-day mortality, hospitalization, ICU admission, mechanical ventilation, or renal replacement therapy in Danish individuals who tested positive for SARS-CoV-2 [67]. In one study, veteran patients were matched via propensity scores, which resolved the differences in age, gender, and care assessment needs (CAN) score, and the odds of mortality were then compared; the study involved 35,370 subjects from 2 March 2020 to 13 September 2020 for the 14-day mortality cohort and 32,836 subjects from 2 March 2020 to 28 August 2020 for the 30-day mortality cohort. Among the COVID-19-positive veterans, a pre-existing aspirin prescription was associated with a statistically and clinically significant decrease in overall mortality at 14 days (OR, 0.38; 95% CI, 0.32–0.46) and 30 days (OR, 0.38; 95% CI, 0.33–0.45). Pre-diagnosis aspirin prescription was strongly associated with decreased mortality rates for veterans diagnosed with COVID-19 [68]. Early aspirin prescription may be associated with lower odds of in-hospital mortality among hospitalized patients with moderate COVID-19 in 2,446,650 COVID-19–positive patients. The cohort screened 189,287 who were hospitalized and 112,269 who met the study inclusion criteria. The 28-day in-hospital mortality rate was substantially lower for those who received aspirin. Patients who received early aspirin did not have higher gastrointestinal hemorrhage, cerebral hemorrhage, or blood transfusion rates. The rate of pulmonary embolism, but not deep vein thrombosis, was also significantly lower in patients who received aspirin. The composite of hemorrhagic complications did not occur more often in those receiving aspirin. The subgroups of patients older than 60 years and patients with comorbidities appeared to benefit from early aspirin use [52]. Aspirin use is associated with decreased mechanical ventilation, ICU admission, and in-hospital mortality in hospitalized COVID-19 patients.

### 3.3. The Acetylation Properties of Dapsone Competitively Anticatalyze COVID-19 Exacerbations

Dapsone (4,4’-diaminodiphenyl sulfone, DDS) is a small molecule with anti-inflammatory, immunosuppressive, antibacterial, and antibiotic properties. Dapsone passes through the blood–brain barrier (BBB) [69,70]. DDS undergoes acetylation and is converted to monoacetyldapsone (MADDS) in a polymorphic fashion by the same enzyme system that acetylates isoniazid and sulfamethazine in humans. MADDS is rapidly deacetylated in humans, and constant plasma ratios of acetylated dapsone are found after taking dapsone [71]. Acetylation contributes to cGAS activity control and provides a potential therapy for treating DNA-mediated autoimmune diseases [49]. cGAS activates downstream IRF3 and NF-κB signaling via STING [54], and both are involved in IFN production [55]. NF-κB can adjust the expression of some interferon-stimulated genes (ISGs) [56]. Dapsone may also deter the activation of both IRF3 and NF-κB by targeting cGAS as a definite cGAS inhibitor. Dapsone has been linked to regulating mild cognitive impairment (MCI), Alzheimer’s disease [72,73,74], and SARS-CoV-2–associated ARDS [40].

### 3.4. Only Dapsone Treats SARS-CoV-2 Exacerbated Acute Respiratory Distress Syndrome

Kanwar et al. administered standard treatments plus dapsone for COVID-19 ARDS patients in the intensive care unit (ICU) from 21 December 2020 to 2021. The treatments were administered to patients with decreased FIO_2_ (fraction of inspired oxygen) and no further progression of hypoxia. The results were statistically significant according to the chi-square test and Fisher’s exact test for the criteria for the effectiveness of dapsone [40,75].

Statistics 1: Chi-square test for ARDS onset and mortality

A chi-square test was performed to analyze the mortality relationship of ARDS onset with dapsone and without dapsone. The chi-square statistic was 5.81, and the *p* value was 0.016 (significant at *p* < 0.05). The group with ARDS onset treated with dapsone was more likely to survive than those with ARDS onset not treated with dapsone.

Statistics 2: Chi-square statistics and Fisher’s exact test

The comparison was made, assuming only the decreased FIO_2_ was significant in the entire dapsone-administered (+) and nonprescribed (–) groups, which applied to only the ARDS-onset stage (Table 1).

Statistics 3: Dapsone offers benefits in the overall complete analysis set

In the complete overall analysis, the median age was 64 years, 58% were men, and 42% were women. The most common risk factors for deteriorating hypoxia and death were CRP data over 150 (in 45%) and oxygen requiring >10 L/min on admission (37%). By specific measures such as initial CRP and oxygen, the patients in the dapsone group were not less sick than those in the control group. Of 30 patients, 16 with dapsone were discharged home, requiring less than 8 L oxygen/min—mostly not more than 5 L oxygen/min; only 6 of 30 patients without dapsone were discharged home (*p* < 0.001). This analysis showed a lower risk of death or discharge to long-term acute care hospitals (RR = 0.52, 95% CI: 0.32 to 0.84) and a higher likelihood of discharge home (RR = 2.7, 95% CI: 1.2 to 5.9) with dapsone compared with those receiving the usual standard of care without dapsone [75].

### 3.5. Dapsone Treatment Mechanism

Dapsone noncovalently binds to the minor groove of DNA, and the dapsone–DNA complex may disrupt DNA replication, repair, transcription, and recombination in SARS-CoV-2 inflammasomes. Dapsone can physically obstruct DNA replication in inflammasomes [40,76]. Therefore, we defined dapsone as a typical anticatalytic medicine. The inflammasome pathway constitutes the primary mechanistic insight into the inextricable relationship between the COVID-19 inflammasome response, spike protein-related diseases, prion-like diseases, and subsequent hematological malignancy.

Dapsone hypersensitivity syndrome is a severe idiosyncratic drug reaction characterized by the clinical triad of fever, rash, and systemic involvement. HLA-B*13:01 was described with a 99.8% negative predictive value and a 7.8% positive predictive value as a risk factor among Chinese patients for dapsone hypersensitivity in 2013 [77]. HLA-B*13:01 is comparatively absent among Europeans and Africans [78]. The affinity of dapsone binding to HLA-B*13:01 is greater than that of HLA-B*13:02 and binds to HLA-B*13:01 more strongly [79,80]. CD8+ clones displayed an HLA-B*13:01-restricted pattern of activation. Dapsone activates specific T cells from hypersensitive patients expressing the risk allele HLA-B*13:01. HLA-B*13:01-CD8+ T cells (cytotoxic T lymphocytes) induce a dapsone-responsive immune response [81]. In a complete and detailed analysis performed with data provided by 98 countries, Leite et al. [82] reported HLA-B*13:01 as a protective allele. They identified several loci related to mortality and the strong association of polymorphisms of the cytokines IL-6, IL-10, and IL-12B [82]. This allele is expressed preferentially in Asiatic populations and interconnected with dapsone-induced hypersensitivity reactions [83].

Dapsone as a supplement was used for the optimum second-line treatment of immune thrombocytopenia [84]. Dapsone can reduce the local expression of mRNA transcripts encoding inflammation-related molecules, including endothelin-1, macrophage inflammatory protein-1-alpha, and transforming growth factor-beta. Dapsone decreased the paraquat-induced generation of superoxide anions in mouse lung fibroblasts [85]. The administration of dapsone reversed the alterations induced by doxorubicin in the serum levels of CK-MB (creatine kinase-MB fraction), electrocardiographic parameters, papillary muscle contractility, and excitation: the measurement of malondialdehyde, superoxide dismutase, and TNF-α levels in tissue indicated that dapsone significantly reduced oxidative stress, consistent with histopathological analysis [72]. Dapsone prevents ischemic injury, inhibits apoptosis, and shows functional improvement in postischemia. It represses the proapoptotic proteins JNK (c-Jun N-terminal kinases), PTEN (phosphatase and tensin homologue), calpain, and caspase-3 in cerebral ischemia. It activates the prosurvival protein BDNF (brain-derived neurotrophic factor) [86]. Dapsone can also protect microvascular integrity from high-fat diet-induced LDL (low-density lipoprotein) oxidation [87]. Dapsone in acetic acid-induced colitis in rats reduced the acetic acid-induced inflammatory response in rat colon tissue by inhibiting the NF-kB signaling pathway [88]. The microbiota is vital for immune homeostasis, providing a competitive barrier to viral infection. The microbiota-driven tonic IFN-I response depended on the second messenger cGAS–STING but not Toll-like receptor signaling. DNA-containing membrane vesicles from the gut microbiota were found in circulation. They promoted the clearance of both DNA (herpes simplex virus type 1) and RNA (vesicular stomatitis virus) viruses in a GAS–STING–IFN-I axis [89]. IFN-I–activated microglia and other brain cells arise and expand with amyloidosis, in which pre- and post-synaptic loss are IFN-I–dependent and mediated by different cell types, and IFN-I signaling in neural cells promotes plaque accumulation [90]. However, we found dapsone as a preventive, therapeutic option for exacerbated AD and COVID-19 ARDS [40,72], and an inhibitor in the GAS–STING–IFN-I axis [91].

### 3.6. Repurposing Drugs That Do Not Treat COVID-19 Type-1 Interferonopathy

The use of antidepressants, such as selective serotonin reuptake inhibitor (SSRI) fluvoxamine, could prevent clinical deterioration in early stage COVID-19 outpatients [92,93]. However, fluvoxamine with metformin and ivermectin cannot prevent the occurrence of hypoxemia, an emergency department visit, hospitalization, or death associated with COVID-19 [94]. It inhibits SARS-CoV-2 replication through ER induction stress and innate immune responses such as cannabidiol [95]. Sensitizing cells to ER may mediate the actions of reactive oxygen species (ROS)-metabolizing ISGs [96]. Fluvoxamine might not use the pathway to anticatalyze type-1 interferonopathy.

The genome-wide DNA methylation profiles of severe COVID-19 cases revealed increased methylation of IFN-related genes, while inflammatory genes were hypomethylated [97]. An ISG expression signature is a hallmark of interferonopathies and other autoimmune diseases. The combined inhibition of histone deacetylases (HDAC) and bromodomain-containing protein 4 (BRD4) resolved the aberrant ISG expression detected in cells derived from patients with two inherited interferonopathies [98] (Table 2).

Clinical trials of COVID-19 vaccines found high vaccine efficacy, and observational studies evaluated vaccine effectiveness in several countries [126,127]. Multiple variants of concern have emerged, such as alpha (B.1.1.7 and Q lineages), beta (B.1.351 and descendent lineages), gamma (P.1 and descendent lineages), epsilon (B.1.427 and B.1.429), Eta (B.1.525), Iota (B.1.526), kappa (B.1.617.1 and B.1.617.3), Mu (B.1.621 and B.1.621.1), zeta (P.2), and, more recently, Omicron (BA.1) and its submicron subvariants (BA.1.1, BA.2, and BA.3), while other receptor-binding domain mutants have also emerged [128]. While evolving and developing new vaccines for each variant is expensive and time-consuming, an urgent need remains to reliably identify and block the ARDS pathological process in critical patient scenarios. We aim to propose the pathophysiology of COVID-19 and provide a treatment that can endure until a vaccine is developed.

## 4. Conclusions

While an immune response is necessary for clearing SARS-CoV-2 infection and establishing immunological memory to combat reinfection, hyperinflammatory responses have also been shown to underlie the pathology of exacerbated COVID-19. COVID-19 therapeutic interventions could have regulated and anticatalyzed immune responses to SARS-CoV-2 in multiple ways. Anticatalytic medicines with acetylating capacity can treat COVID-19.

## Figures and Tables

**Table 1:** Effects of dapsone as applicable to COVID-19 ARDS onset.

Dapsone-Administered (+)/Nonprescribed (–)	Decreased FIO_2_	Others	Row Totals	Dapsone-Administered (+) Group	Decreased FIO_2_ + No Progressive ARDS	Progressive ARDS	Row Totals
Dapsone (+) onset *^1^	7	1	8	Dapsone (+) onset *^2^ + Aggravated *^3^	17	3	20
Dapsone (–) onset	8	12	20	Dapsone (+) severe *^4^	0	2	2
Totals	15	13	28	Total	17	5	22

Chi-square statistic: 5.18; *p* value: 0.023. The result is significant at *p* < 0.05. *1: FIO_2_ (fraction of inspired oxygen) necessity via simple nasal cannulation of up to 15 L/min (reproduced from Kanwar B, Lee CJ, Lee JH. Specific Treatment Exists for SARS-CoV-2 ARDS); Fisher’s exact test statistic value: 0.043 (significant at *p* < 0.05); *2: FIO_2_ (fraction of inspired oxygen) requirement via simple nasal cannulation of up to 15 L/min; *3: FIO_2_ administered via an HFNC (high-flow nasal cannula) of 95–100% and/or BiPAP (bilevel positive airway pressure); *4: requiring mechanical ventilation (reproduced from [40].

**Table 2:** Epigenetically upregulated receptors/mediators associated with SARS-CoV-2 entry.

Factor	Receptor Mediator	Role in COVID-19	Histone Marks in ^1^ *mdig*^+^ Epithelial Cells	Ref.
Aging	ACE2	Age-dependent DNA methylation was observed close to the transcription start site of the ACE2 gene in airway epithelial cells. Histone modifications and the levels of histone proteins change during aging, dramatically influencing chromatin compaction and gene expression.	↓ ^3^ H3K9me3 ↓^3^ H3K27me3 ↑^3^ H3K36me3 ↑^3^ H4K20me3	[99] [100] [101] [102]
	DNA methylation	^*^↑ methylation of IFN-related genes. ^**^↓ Inflammatory genes hypomethylated.		[97]
Smoking		Epigenetic mechanisms alter the common transcriptional bridge between smoking and COVID-19 by trimethylation of particular lysine (K) residues at H3 and H4 histones.	↑ H3K4me3 ↓ H3K9me3 ↓ H3K27me3 ↓ H4K20me3	[103]
	ACE2	The primary receptor mediating SARS-CoV-2 entry, the expression of ACE2 is maintained, if not upregulated, due to the arsenic-induced impaired activity of ^2^ EZH2.	↓ ^3^ H3K27me3	[104] [105] [106]
	^4^ NRP1	They are highly expressed in the respiratory tract epithelial cells; NRP1 binds the S1 segment of the SARS-CoV-2 spike protein following its cleavage by furin.	↑ ^3^ H3K4me3 ↓ ^3^ H3K9me3	[104] [107]
	NRP2	Similar to NRP1.	↑ ^3^ H3K4me3 ↓ ^3^ H3K9me3 ↓ ^3^ H3K27me3	[104] [108]
	^5^ AT1R	It facilitates SARS-CoV-2 entry through receptor-mediated endocytosis of ACE2-S complex following the interaction of viral spike protein with soluble ACE2.	↓ ^3^ H3K9me3	[104] [109] [110]
	^6^ CTSD	Potentially facilitates SARS-CoV-2 entry through positive regulation of furin through osteopontin.	↓ ^3^ H3K9me3 ↓ ^3^ H3K27me3	[104] [111,112,113]
	^7^ CTSL	Elevated in the serum of COVID-19 patients, CTSL mediates viral entry by participating in the cleavage of the viral spike protein.	↑ ^3^ H3K4me3 ↓ ^3^ H3K9me3 ↓ ^3^ H3K27me3	[104] [114]
	^8^ PTGER2-4	Upregulation of PGE2 receptors might potentiate the positive regulatory effect of PGE2 on ACE2 and TMPRSS2, facilitating SARS-CoV-2 entry.	↑ ^3^ H3K4me3	[104] [115,116]
	^9^ SLC6A20/SIT1	Positively regulated by ACE2, SLC6A20/SIT1 is suspected of interacting with ACE2 and enhancing its activity reciprocally.	↓ ^3^ H3K27me3	[104] [117,118]
	IL-6	Present in high levels in the serum of COVID-19 patients, IL-6 is speculated to enhance viral entry by activating the AT1R signaling cascade.	↓ ^3^ H3K27me3	[110,119,120,121]
^10^ ISG	^11^ HDAC	An ISG expression signature is a hallmark of interferonopathies and other autoimmune diseases. The combined inhibition of HDAC1/2 and ^12^BRD4 resolved the aberrant ISG expression detected in cells derived from patients with two inherited interferonopathies [98].	↑ ^3^ H3K36me3	[101]
	^13^MDA5	MDA5 preferentially binds negative-strand SARS-CoV-2 RNA. Active viral replication is required for triggering MDA5 activation. Type I/III IFN induction by SARS-CoV-2 relies on MDA5.	In study	[122] [123]
	^14^ RIG-I	RIG-I binds preferentially blunt-ended short dsRNA bearing a 5’ triphosphate moiety such as the ^15^IAV (sub)genomic panhandle structure and exerts IFN-independent antiviral activity by competing with the viral polymerase for binding to the 3’ untranslated region (UTR) of the SARS-CoV-2 genomic RNA. Both MDA5 and RIG-I may contribute to SARS-CoV-2 restriction in a temporal manner.	In study	[124] [125]

^1^ The heterozygous mdig knockout (mdig^+^), ^2^ enhancer of zeste homolog 2 (EZH2)—a histone-lysine N-methyltransferase enzyme encoded by the EZH2 gene that joins in histone methylation and transcriptional repression. EZH2 catalyzes the addition of methyl groups to histone H3 at lysine 27 using the cofactor S-adenosyl-L-methionine. ^*^ Increased, ^**^ Decreased, ^3^ Neuropilin (NRP) ^4^ packaging protein histone H3 or H4 series (H3K27me3, H3K4me3, H3K9me3, H4K20me3)—an epigenetic modification to the DNA, ^3^ neuropilin (NRP), ^5^ angiotensin II type 1 receptor (AT1R), ^6^ CTSD (cathepsin D) encoded by the CTSD gene. This gene encodes a lysosomal aspartyl protease arranged in a protein dimer of disulfide-linked heavy and light chains produced from a single protein precursor. ^7^ CTSL (cathepsin L) is a cysteine cathepsin, a lysosomal cysteine protease that plays a major role in intracellular protein catabolism. ^8^ Prostaglandin E receptor (PTGER), a prostaglandin receptor subtype, is a protein-coding gene. ^9^ Solute carrier family 6 member 20 (SLC6A20)/sodium-dependent amino transporter 1 (SIT1)—SIT1, the product of the slc6a20 gene. ^10^ Interferon-stimulated gene (ISG). ^11^ Histone deacetylases (HDAC) HDAC activity provides a required positive function for IFN-stimulated gene (ISG) expression. ^12^ Bromodomain-containing protein 4 (BRD4) is a protein encoded by the BRD4 gene. Bromodomain-containing protein 4 is a protein that is encoded by the BRD4 gene in humans. ^13^ Melanoma differentiation-associated protein 5 (MDA5). ^14^ Retinoic acid-inducible gene I (RIG-1). ^15^ Influenza A virus (IAV) (reproduced from SHIRVALILOO, M. 2022. The unfavorable clinical outcome of COVID-19 in smokers is mediated by H3K4me3, H3K9me3, and H3K27me3 histone marks. *Epigenomics*, 14, 153–162).

## Data Availability

No new data were created or analyzed in this study. Data sharing is not applicable to this article.

## Abbreviations

ACE	Angiotensin-converting enzyme
AD	Alzheimer’s disease
AESI	Adverse events of special interest
AGS	Aicardi–Goutières syndrome
AMI	Acute myocardial infarction
AMI I/R injury	AMI ischemia–reperfusion injury
ARDS	Acute respiratory distress syndrome
ASC	Apoptosis-associated speck-like protein containing a CARD
ATF4	Activated parkin via protein kinase RNA-like endoplasmic reticulum kinase-activating transcription factor 4
BDNF	Brain-derived neurotrophic factor
BiPAP	Bilevel positive airway pressure
BV-2	A type of microglial cell derived from C57/BL6 mice
CAPS	Cryopyrin-associated periodic syndromes
CARD	Caspase activation and recruitment domain
CCNE2	Essential for the control of the cell cycle at the late G1 and early S phases; belongs to the cyclin family
CCR2	C–C motif chemokine receptor 2
CH	Clonal hematopoiesis, hematopoietic stem and progenitor cells
CI	Confidence interval
CK-MB	Creatine kinase-MB fraction
COPD	Chronic obstructive pulmonary disease
COX-2	Cyclooxygenase 2
CRP	C-reactive protein
CRS	Cytokine release syndrome
Cyclin E2	Cyclin E2 is a protein that in humans is encoded by the CCNE2 gene
DDS	4,4′-Diaminodiphenyl sulfone (dapsone)
DIC	Disseminated intravascular coagulation
ECG	Electrocardiogram
G6PDH	Glucose-6-phosphate dehydrogenase
HLA	Human leukocyte antigen
HLA-DRB1	Major histocompatibility complex, class II, DR beta 1
ICU	Intensive care unit
IFN	Interferon
IFNAR2	Interferon-alpha and beta receptor subunit 2
IL	Interleukin
IL-1β	Interleukin-1 beta
IMV	intensive mechanical ventilation
IRF3	Interferon regulatory factor 3
JNK	Jun N-terminal kinases
LDH	Lactate dehydrogenase
LDL	Low-density lipoprotein
LL	Lepromatous leprosy
MADDS	Monoacetyldapsone
MAPK	Mitogen-activated protein kinase
MCI	Mild cognitive impairment
MHC	Major histocompatibility complex
MIS-C/A	Multisystem inflammation syndrome in children and adults
MPO	Myeloperoxidase
NOD2	Nucleotide-binding oligomerization domain containing 2
mRNA	Messenger RNA
mtDNA	Mitochondrial DNA
NACHT	Domain conserved in NAIP, CIITA, HET-E, and TP1
NF-κB	nuclear factor kappa-light-chain-enhancer of activated B cells
NLRP3	NOD-, LRR-, and pyrin domain-containing protein 3
PAI-1	Plasminogen activator inhibitor-1
PAMPs	Pathogen-associated molecular patterns
PBMCs	Human peripheral blood mononuclear cells
PD	Parkinson’s disease
PEDF	Pigment epithelium-derived factor
PEDFR/iPLA2	PEDF/calcium-independent phospholipase A2
Phospho-p65	Anti-phospho-NFkB p65 (Ser536) monoclonal antibody (T.849.2)
Phospho-IκBα	Phospho-IκBα (Ser32/36) (5A5) mouse mAb #9246
PTGS2	Prostaglandin synthase 2
PTM	Multiple post-translational modification
ROS	Reactive oxygen species
S	Full-length prefusion spike glycoprotein of SARS-CoV-2
S1	SARS-CoV-2 spike protein subunit 1
SCLS	Systemic capillary leak syndrome
RCT	Randomized controlled trial
SOD	Superoxide dismutase
TGF-β	Transforming growth factor-beta
THP-1	A spontaneously immortalized monocyte-like cell line
TNF	Tumor necrosis factor
TLR	Toll-like receptor
TMPRSS2	Transmembrane protease serine subtype 2
TTS	Thrombosis with thrombocytopenia syndrome
TYK2	Tyrosine kinase 2
